# Canine Neuronal Ceroid Lipofuscinosis-like Disorder Associated with Sequence Variants in *AP3B1* and *TRAPPC9*

**DOI:** 10.3390/genes16111370

**Published:** 2025-11-11

**Authors:** Alexander Then, Rebecca Welly, Garrett Bullock, Lucie Chevallier, Martin L. Katz

**Affiliations:** 1 Blå Stjärnans Djursjukhus, SE-43137 Mölndal, Sweden; alexander.then@gmail.com; 2 Faculty of Medicine and Health, School of Medical Sciences, Örebro University, SE-70182 Örebro, Sweden; 3 Canine Genetics Laboratory, Department of Veterinary Pathobiology and Integrative Biomedical Sciences, College of Veterinary Medicine, University of Missouri, Columbia, MO 65211, USA; wellyr@missouri.edu (R.W.); gebkd2@missouri.edu (G.B.); 4 U955—IMRB, Team 10—Biology of the Neuromuscular System, Institut National de la Santé et de la Recherche Médicale, Ecole Nationale Vétérinaire d’Alfort, 94704 Maisons-Alfort, France; lucie.chevallier@vet-alfort.fr

**Keywords:** lysosomal storage disease, lipofuscin, neurodegeneration, magnetic resonance imaging, whole-genome sequencing

## Abstract

Background/Objectives: A Petit Bleu de Gascogne (PBDG) dog presented with a progressive neurological disorder characterized by hind-limb weakness, anxiety and hallucinatory episodes, lip smacking, progressive vision loss, muscle atrophy, and ataxia. Magnetic resonance imaging revealed diffuse brain atrophy. The dog was euthanized at approximately 23 months of age due to the progression of neurological signs. A study was undertaken to identify the molecular genetic basis of the disorder in this dog. Methods: Microscopic analyses were performed to characterize the disease pathology and whole-genome sequencing was performed to identify the molecular genetic basis of the disorder. Results: The proband exhibited pronounced accumulations of autofluorescent intracellular inclusions in the brain, retina, and heart with ultrastructural appearances similar to those of lysosomal storage bodies that accumulate in the neuronal ceroid lipofuscinosis (NCLs), a group of progressive neurodegenerative disorders. Whole-genome sequence analysis of DNA from the proband identified homozygous missense variants in *AP3B1* and *TRAPPC9* that encode proteins involved in sorting and transport of proteins through the Golgi apparatus to lysosomes. Screening of unaffected PBDGs for these variants identified dogs that were homozygous for either variant, but no other dogs that were homozygous for both. Conclusions: These findings raise the possibility that the disease involves the combined influence of the two variants, and that the proteins encoded by these genes interact within the Golgi apparatus to mediate protein sorting and transport to lysosomes. An alteration in this interaction could underlie the NCL-like lysosomal storage disorder observed in the proband.

## 1. Introduction

Dogs of numerous breeds suffer from hereditary progressive neurodegenerative diseases associated with variants in orthologs of genes harboring sequence variants that underlie corresponding disorders in human subjects. Among the most common of these diseases are the neuronal ceroid lipofuscinoses (NCLs). Behavioral signs of canine NCLs usually include progressive impairment of coordination, cognitive decline, seizure activity, changes in behavior, and progressive visual impairment [1]. Variants in at least 13 genes have been associated with different forms of human NCL [2,3,4,5,6]. Canine NCLs have been identified that result from mutations in the orthologs of the majority of these genes [1,7,8,9,10,11]. The NCLs are distinguished from other neurodegenerative disorders with similar clinical signs by the accumulation throughout the central nervous system and other tissues of lysosomal storage bodies with distinctive autofluorescence properties that are similar to those of age pigment (lipofuscin) [2,6,12,13,14,15] combined with progressive brain atrophy. In most cases when progressive neurodegeneration was accompanied by autofluorescent storage body accumulation in dogs, the causal variant has been found to occur in one of the NCL genes [1]. However, accumulation of lipofuscin-like autofluorescent cellular inclusions has also been found to occur in some lysosomal storage diseases not classified as NCLs [16,17,18,19,20,21]. For dogs that exhibit progressive neurological signs and autofluorescent storage body accumulation, whole-genome sequencing (WGS) can be used to screen for potential disease-causing variants in all of the known NCL genes and any in other potential candidate genes. This approach has been used successfully to identify the causal mutations for many canine hereditary neurodegenerative disorders including those classified as NCLs [1,8,9,22,23] and other lysosomal storage diseases [16,18,24,25,26]. A study was undertaken to determine whether a PBDG dog that had exhibited signs suggestive of NCL exhibited NCL-like tissue pathology and to identify the molecular genetic variants associated with the disorder.

## 2. Materials and Methods

### 2.1. Neurological Examination

A neurological examination of the proband, an intact female PBDG, was performed at 22 months of age. This included a complete blood count and blood chemistry analyses and magnetic resonance imaging (MRI) of the proband’s brain. For MRI the dog was sedated with subcutaneously with dexmedetomidine 125 µg/m^2^ (Dexdomitor^®^, injection solution 0.5 mg/mL; Orion Pharma Animal Health, Espoo, Finland), butorphanol 0.27 mg/kg (Dolorex^®^ injection solution 10 mg/mL; MSD Animal Health Sweden AB, Stockholm, Sweden), and intravenously with midazolam 0.2 mg/kg (Midazolam Accord, injection/infusion solution 5 mg/mL; Accord Healthcare AB, Solna, Sweden). MRI was performed with a Toshiba Vantage Elan 1.5-Tesla instrument (Canon Medical Systems Corporation, Otawara, Tochigi, Japan). Acquisitions included T2-weighted fast spin-echo in sagittal, axial, and dorsal planes; axial T2-weighted FLAIR; flow-sensitive black-blood, a susceptibility-weighted sequence analogous to SWI; diffusion-weighted imaging (b = 1000 s/mm^2^) with calculated apparent diffusion coefficient (ADC) maps; and 3D T1-weighted fast gradient-echo before and after gadolinium-based contrast. Gadoteridol was administered intravenously at 0.1 mmol/kg (ProHance^®^, injection solution 279.3 mg/mL [0.5 mmol/mL]; Bracco Imaging S.p.A., Milan, Italy), corresponding to 0.2 mL/kg. After the examination, atipamezole was administered at a dose of 0.05 mg/kg intramuscularly. During the sedation, blood oxygen saturation and pulse rate were monitored with a fiber-optic pulse oximeter (Model 7500FO; Nonin Medical, Inc., Plymouth, MN, USA). Respiratory motion and rate were monitored using a pneumatic respiratory bellows (belt) integrated with the MRI system (Canon Medical Systems Corporation, Otawara, Tochigi, Japan). The MRI protocol met the requirements of the guidelines from the American Association of Veterinary Radiologists (AAVR). MR images were reviewed and interpreted by both a board-certified veterinary radiologist (European College of Veterinary Diagnostic Imaging) and a board-certified veterinary neurologist (European College of Veterinary Neurology).

### 2.2. Tissue Collection, Processing, and Microscopic Analyses

The proband was humanely euthanized at 22 months and 21 days of age due to the progression of neurological signs. Premedication consisted of acepromazine 0.06 mg/kg subcutaneously (Plegicil^®^ injection solution 10 mg/mL; Pharmaxim AB, Landskrona, Sweden) and butorphanol 0.35 mg/kg subcutaneously (Dolorex^®^ injection solution 10 mg/mL; MSD Animal Health Sweden AB, Stockholm, Sweden). Approximately 15 min later, euthanasia was performed via intravenous injection of sodium pentobarbital 100 mg/kg into the cephalic vein (Allfatal^®^ injection solution 100 mg/mL; Omnidea AB, Stockholm, Sweden). Death was confirmed by absence of cardiac activity and corneal reflexes. Following euthanasia, brain, eye, and heart tissues were collected for microscopic analyses as described previously [18]. One eye was fixed in 2.5% cacodylate-buffered glutaraldehyde, and the other eye was fixed in “Immuno” fixative (cacodylate-buffered 3.5% paraformaldehyde and 0.05% glutaraldehyde). Slices of the cerebral cortex parietal lobe, cerebellar cortex, and heart ventricular wall were fixed in the Immuno fixative and in EM fixative (cacodylate-buffered 2.0% glutaraldehyde, 1.12% formaldehyde). The fixed tissues were prepared for light and electron microscopic evaluations and for immunohistochemistry, as described previously [16,18]. All microscopic evaluations of the retina were performed on samples obtained from the region within 1 cm of the optic nerve head along the superior-inferior midline.

For assessment of tissue autofluorescence, slices of the Immuno-fixed tissues were embedded in an OCT medium (Tissue-Tek, Sakura Finetek, Torrance, CA, USA) and cryo-sectioned at a thickness of 8 mm. The unstained sections were examined for autofluorescence using a Zeiss Axiophot microscope (Carl Zeiss AG, Oberkochen, Germany) equipped with a Prior Lumen 200 light source (Prior Scientific Instruments Ltd., Cambridge, UK), a 395–440 nm bandpass excitation filter, an FT 460 dichromatic beam splitter, and an LP 470 barrier filter. A 515 nm barrier filter was also placed in the emission light path. Fluorescence images were acquired using a Zeiss Neofluor 40× objective with a numerical aperture of 0.75 and an Olympus DP72 digital camera (Olympus Corporation, Tokyo, Japan).

For immunohistochemistry, slices of both Immuno-fixed tissues were paraffin-embedded, and sections were immunostained for localization of glial fibrillary acidic protein (GFAP), ionized calcium-binding adapter molecule 1 (Iba1), and mitochondrial ATP synthase subunit c. Primary antibodies used for immunolocalization were Abcam anti-mitochondrial ATP synthase subunit c primary antibody (cat. No. ab180149), Agilent Dako anti-GFAP primary antibody (cat. No. Z0334), and Fujifilm Wako anti-Iba1 primary antibody (cat. No. 019-19741). Immunohistochemical staining was performed as described previously [18,27]. Adjacent tissue sections were subjected to the same immunolabeling protocol except with non-immune serum replacing the primary antibodies. No labeling was observed in these sections, validating the specificity of the immunolabeling.

EM-fixed cerebral cortex, cerebellar cortex, and retina samples were also processed for electron microscopy. This included secondary fixation in osmium tetroxide and embedding in epoxy resin. Sections of selected areas from each tissue were cut at thicknesses of 80 to 90 nm, mounted on copper grids, and stained with lead citrate and uranyl acetate. The sections were examined with a JEOL 1400 transmission electron microscope (JEOL Ltd., Tokyo, Japan) equipped with Gatan digital camera (Gatan, Inc., Pleasanton, CA, USA).

All microscopic analyses were performed by an expert on canine NCL histopathology (MLK) using established procedures [11,12,13,24].

### 2.3. Molecular Genetic Analyses

**Figure 1:**
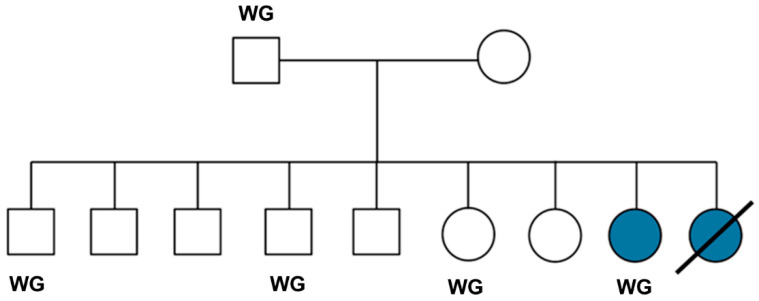
Pedigree of the proband’s litter. Filled symbols indicate the female proband and one other female dog that exhibited signs similar to those of the proband (symbol with slash-through). The latter dog died as a result of the neurological disorder and no samples were obtained from it. DNA samples for whole-genome sequence analyses (WG) were obtained from the proband, three unaffected littermates, and the unaffected sire of the litter. Open circles represent unaffected females, open squares represent unaffected males.

The proband was one of nine littermates, one of which had died after exhibiting signs similar to those of the proband (Figure 1). Genomic DNA from the healthy sire of the litter, from the proband, and from 3 healthy littermates as well as 6 other PBDG dogs that did not exhibit any neurological abnormalities was prepared from EDTA-anticoagulated blood as previously described [28]. A littermate of the proband had already been euthanized due to disease progression at the owner’s request prior to the proband presenting to us. No blood or tissue samples had been saved from this dog that would have enabled us to perform analyses similar to those performed on the proband. We only learned of the deceased affected littermate after the study was initiated. We were unable to recruit DNA samples from some of the other littermates and the dam of the litter. These dogs were all from Sweden. DNA was also isolated from buccal cells of 32 additional PBDG dogs from France that were not closely related to the proband, using the Maxwell^®^ 16 Buccal Swab LEV DNA Purification Kit and the Maxwell^®^ 16 Instrument (Promega, Dübendorf, Switzerland). The DNA samples from the proband, the sire and 3 unaffected littermates were submitted to the University of Missouri Genomics Technology Core Facility for library preparation and 2 × 150 bp paired-end sequencing on an Illumina NovaSeq 6000 sequencer. A previously described data-processing pipeline was used to align the sequence reads to a current canine reference genome assembly (Dog10K_Boxer_Tasha) and to analyze them in conjunction with reads from 383 other canine whole-genome sequences previously generated by the University of Missouri Canine Genetics Laboratory that were used as additional unaffected controls [29]. The control sequences were generated from both healthy dogs and dogs with a variety of hereditary disorders, including dogs with various forms of NCL. The whole-genome sequences from the control cohort have been deposited in the NCBI Sequence Read Archive (SRA) (https://trace.ncbi.nlm.nih.gov/Traces/sra/sra.cgi; accessed on 10 November 2025) [29]. The whole-genome sequences from the PBDG dogs were also deposited in the SRA. The BioSample IDs are SAMN39309475 (proband), SAMN52585581 (sire), SAMN52585580 (littermate 1), SAMN52585582 (littermate 2), and SAMN52585583 (littermate 3). The BioSample IDs for the dogs utilized for this study are listed in Supplementary File S1.

Allelic discrimination assays were used to genotype all of the PBDG dogs for candidate variants in *AP3B1* and *TRAPPC9*. For the *AP3B1* assay, the sequences of the PCR primers were 5′-ACCCAGTATCCACACCAGTTGTA-3′ and 5′-TGACAAGTTTAAACCTTCAAGATCAGCTA-3′. The competing probes’ sequences were 5′-VIC-AGAGCTGGTGTGGGAAG-NFQ-3′ and 5′-FAM-AAGAGCTGGTATGGGAAG-NFQ-3′. For the *TRAPPC9* assay, the sequences of the PCR primers were 5′-GGAGAAGTTCCACGGACATGT-3′ and 5′-CCAGGCCGGGATGCT-3′. The competing probes’ sequences were 5′-VIC-CTCCTTGGTACATTACC-NFQ-3′ and 5′-FAM- CTCCTTGGTACGTTACC-NFQ-3′. The PBDGs were also screened for candidate variants in ATP6AP1L and APLP2. The ATP6AP1L allelic discrimination assay primer sequences were 5′-GCAAGAATCTGGTCCAAAGTTAGTGA-3′ and 5′-TGCTTGGTTTGAAGAAACTGATTCATTT-3′. The competing probes’ sequences were 5′-VIC-CTTGCTTAGTTTTTCACTTAT-NGQ-3′ and 5′-FAM-CTTGCTTAGTTTTCACTTAT-NFQ-3′. Samples were screened for APLP2 by a 2-step allelic discrimination method previously described [30]. Briefly, the first step was PCR amplification with primers 5′-AAACCAGCTCAACTCACGGT-3′ and 5′-CAGCATGGCAGCTTGTTGAC-3′ using Promega GoTaq Flexi DNA Polymerase (Madison, WI, USA; Promega Catalog #: M8295). Cycling parameters consisted of an initial denaturing at 95 °C for 2 min, followed by 40 cycles of denaturing at 95 °C for 15 s, annealing at 61 °C for 15 s, extension at 72 °C for 30 s, and a final extension at 72 °C for 2 min. The completed amplicon was then used as the DNA template for the second step employing a custom TaqMan SNP Genotyping Assay (ThermoFisher, Walthan, MS, USA; Catalog Assay ID: ANT2WH7). The custom-designed assay probed the context sequence 5′-TTCCTCTTCA(TCC/-)TCCTCCTCCT-3′ using VIC and FAM reporter dyes to amplify the reference and variant alleles, respectively. Chi-square analysis was used to assess the genotype-phenotype association with the cohort of 426 dogs that were evaluated in this study.

All aspects of these studies were approved by the relevant Institutional Review Boards (see Institutional Review Board Statement section below).

## 3. Results

### 3.1. Disease Phenotype

**Figure 2:**
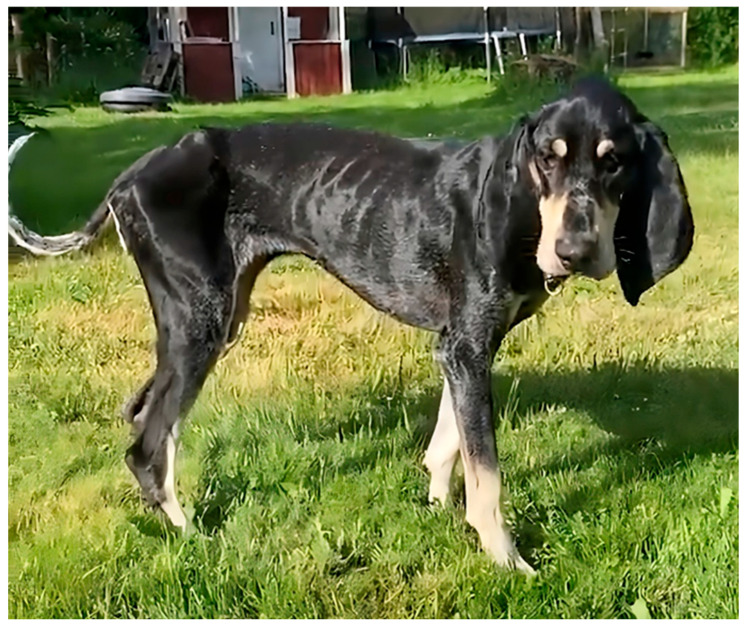
Photograph of the proband with generalized muscular atrophy in an early stage of the disease progression.

The proband was an intact purebred female PBDG (Figure 2) that was acquired at 8 weeks of age by the owners from a PBDG breeder. The dog received routine vaccinations and at the time of adoption did not exhibit any health issues other than several persistent deciduous teeth; these were surgically removed. Starting at approximately 5 months of age, the proband exhibited intermittent brief episodes of head bobbing. At 12 months the proband developed a pronounced loss of appetite and by 18 months became quite emaciated. She became resistant to walking outside during this period. The inappetence was reversed with a short course of oral administration of omeprazole, although the proband’s muscles remained atrophic despite eating a sufficient amount of dry food. A complete blood chemistry analysis did not identify any abnormalities at this time. Over the following months, the proband developed hind-limb weakness, behavioral signs that included anxiety and hallucinatory episodes, lip smacking, and progressive vision loss, initially only in dim light but progressing to include visual impairment in bright light conditions. The dog became progressively more ataxic and intolerant of grooming or handling.

When examined at 22 months of age the dog exhibited ataxia and muscle atrophy of all four limbs with a prominent hypermetric front limb gait. Her balance was severely impaired, and head tremors were present during both intentional movement and when at rest. Paw placement (conscious proprioception) was normal in all four limbs. Hopping responses were decreased in all four limbs. She was hypersensitive to tactile stimuli of the skin and exhibited flight behavior and a reduced menace response. Routine blood chemistry and blood cell analyses did not show any abnormalities (Supplementary File S2). Her body weight was 16.4 kg, her body condition score was 2/9 [31,32] and her body surface area was 0.652 m^2^.

**Figure 3:**
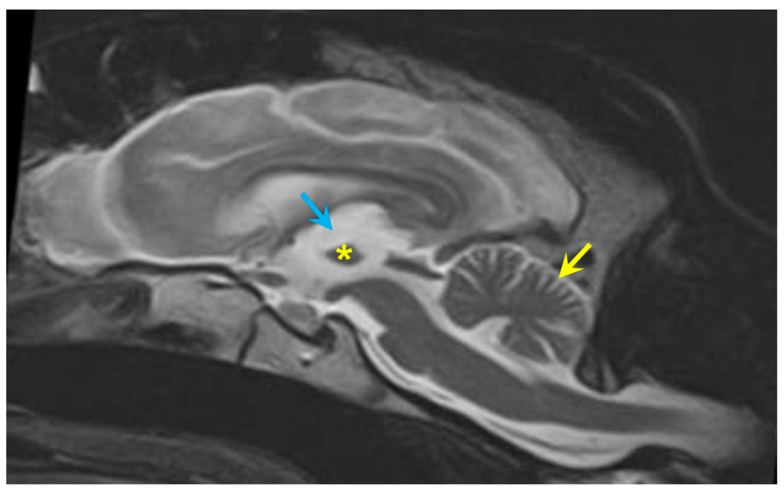
A sagittal T2-weighted MR image of the proband’s brain shows an irregular and small interthalamic adhesion (asterisk) increased prominence of the cerebellar folia (yellow arrow), and an enlarged third ventricle (blue arrow).

**Figure 4:**
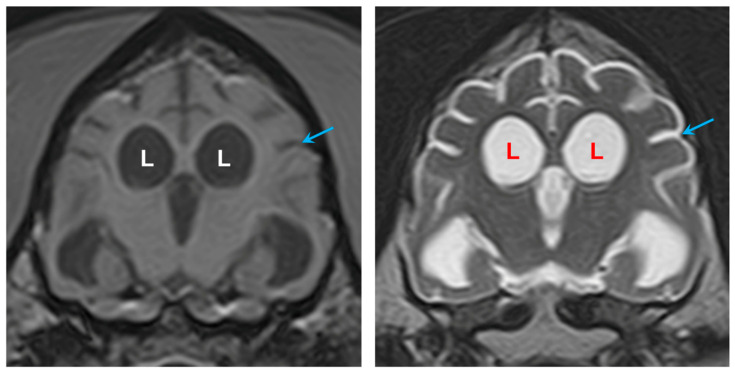
Transverse T1-weighted image (**left**), and at the same level a transverse T2-weighted image (**right**). The lateral ventricles (L) were enlarged, and the cortical sulci (arrows) were wider than normal.

**Figure 5:**
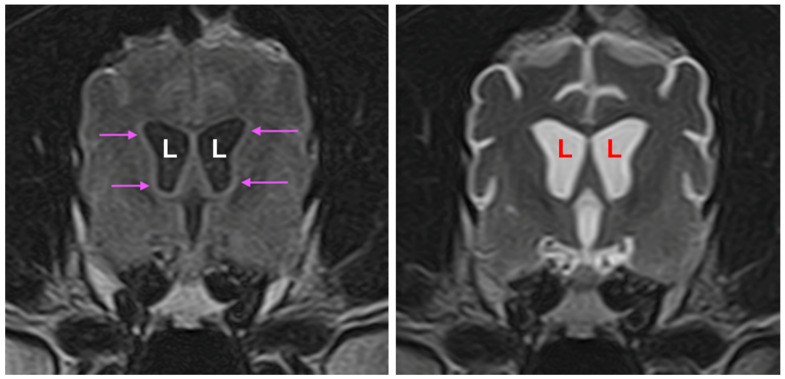
Transverse FLAIR image showing hyperintense areas (arrows) surrounding the enlarged lateral ventricles (L) (**left**). Transverse T2-weighted image at the same level (**right**) for comparison that this subtle finding can be difficult to appreciate without FLAIR imaging.

MRI showed moderate, generalized enlargement of the ventricular system with diffuse widening of the cerebral sulci and cerebellar folia; the interthalamic adhesion was reduced in size (Figure 3). Gray–white matter differentiation was poor, and the cerebral cortex appeared diffusely thinned (Figure 3, Figure 4 and Figure 5). Fluid Attenuated Inversion Recovery (FLAIR) imaging showed an area of hyperintensity surrounding the lateral ventricles (Figure 5). There was no evidence of space-occupying lesions and brain symmetry was preserved.

### 3.2. Microscopic Findings

**Figure 6:**
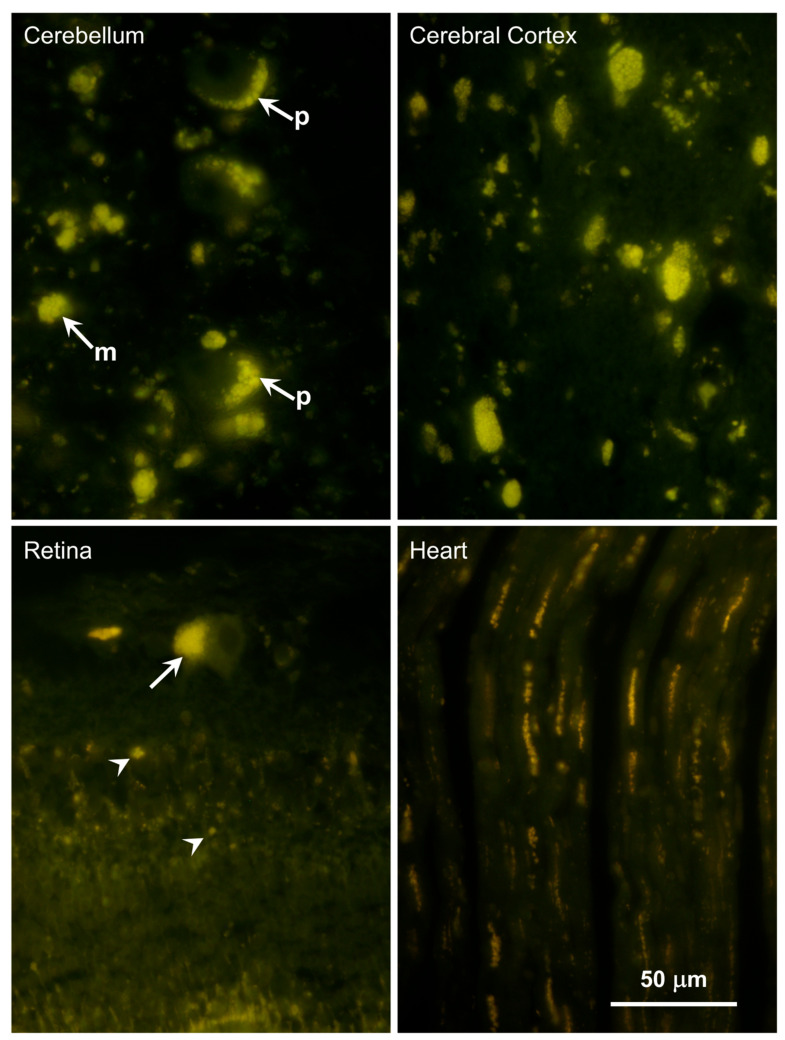
Fluorescence micrographs of unstained cryostat sections of tissues from the proband. In the cerebellum aggregates of autofluorescent inclusions were abundant in the Purkinje cells (p) and in the molecular layer (m). In the cerebral cortex, similar inclusions were present in cells throughout the gray matter. In the retina, the ganglion cells contained aggregates of these inclusions (arrow), and punctate autofluorescent bodies were present in other retinal layers (arrowheads). In cardiac muscle, linear arrays of punctate autofluorescent inclusions were present in the muscle fibers. Bar indicates magnification of all four micrographs.

**Figure 7:**
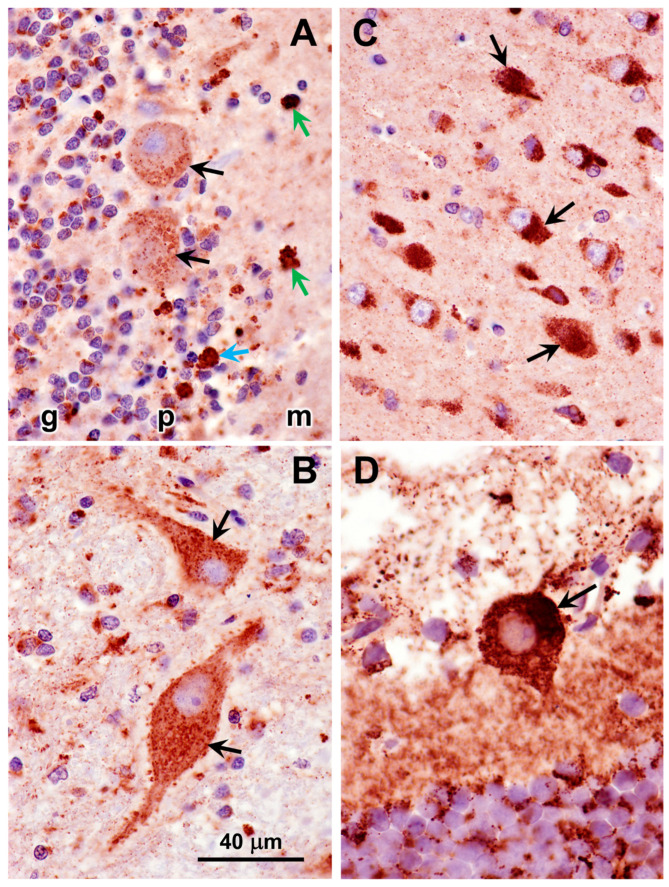
Sections of the cerebellar cortex (**A**,**B**), cerebral cortex (**C**), and retina (**D**) immunostained for localization of subunit c of mitochondrial ATP synthase (red-brown stain). Cerebellar layers: (g) granule cell layer; (p) Purkinje cell layer; (m) molecular layer. In the cerebellum, immunostained inclusions were present in Purkinje cells (black arrows in **A**) in other cells in the Purkinje cell layer (blue arrow in **A**), and in the molecular layer (green arrow in **A**). Large neurons in deeper cerebellar nuclei contained large numbers of immunostained punctate inclusions (arrows in **B**). In the cerebral cortex gray matter, almost all cells contained immunostained inclusions (arrows in **C**). In the retina, the ganglion cells exhibited intense immunostaining (arrow in **D**). Bar in (**B**) indicates magnification for all four micrographs.

**Figure 8:**
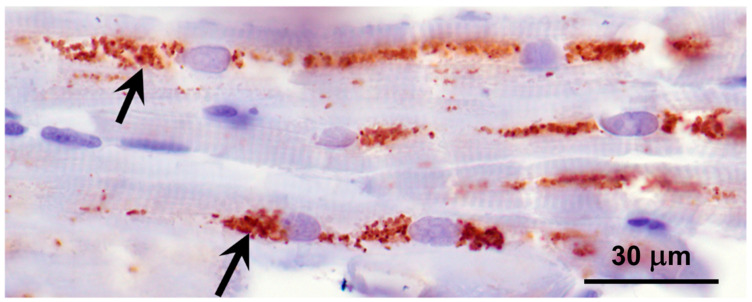
Section of cardiac ventricular wall muscle immunostained for localization of subunit c of mitochondrial ATP synthase (red-brown stain). Aggregates of immunostained inclusions flanked most of the muscle fiber nuclei (arrows).

Abundant autofluorescent inclusions were present in unstained sections of the cerebellar cortex, cerebral cortex gray matter, retina, and cardiac muscle of the proband (Figure 6). The fluorescence spectral properties of these inclusions were similar to those of lysosomal storage bodies that accumulate in the NCL diseases, with yellow to orange emissions when excited with blue light [23,33,34,35,36]. In the cerebellar cortex, the inclusions were present primarily in Purkinje cells and cells in the granular layer. Cells throughout the cerebral cortex gray matter contained large amounts of the autofluorescent material. In the retina, large aggregates of the autofluorescent inclusions were present in the ganglion cells, while individual small inclusions were present in the inner nuclear layer. In the cardiac muscles, the autofluorescent inclusions were arrayed in linear clusters along the long axes of the muscle fibers and exhibited orange emission in contrast to the more yellow emission of the inclusions in the neural tissues. The storage bodies in all of the tissues exhibited strong immunolabeling with an antibody directed against the subunit c protein of mitochondrial ATP synthase (Figure 7 and Figure 8).

**Figure 9:**
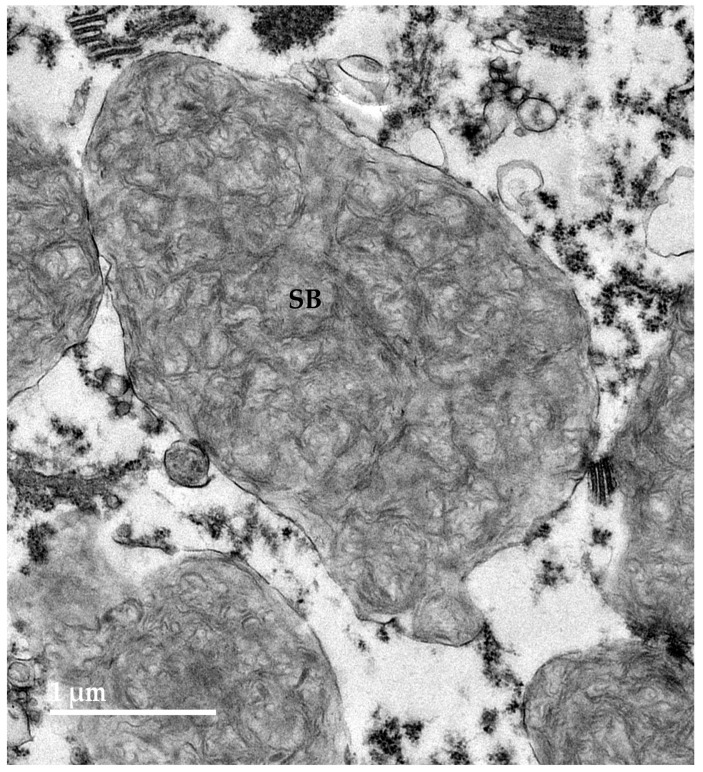
Electron micrograph of a representative storage body (SB) in a cerebellar Purkinje cell of the proband. The storage bodies in all cell types in the cerebellar cortex were similar in appearance.

**Figure 10:**
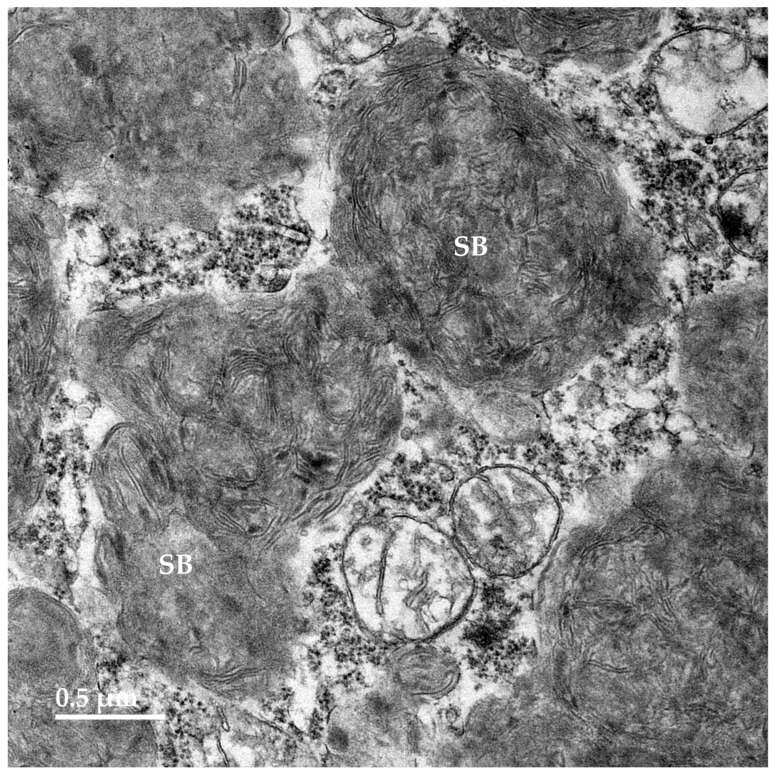
Electron micrograph of storage bodies (SB) in a cerebral cortical neuron of the proband. Most storage bodies in the cells of the cerebral cortex were similar in appearance.

**Figure 11:**
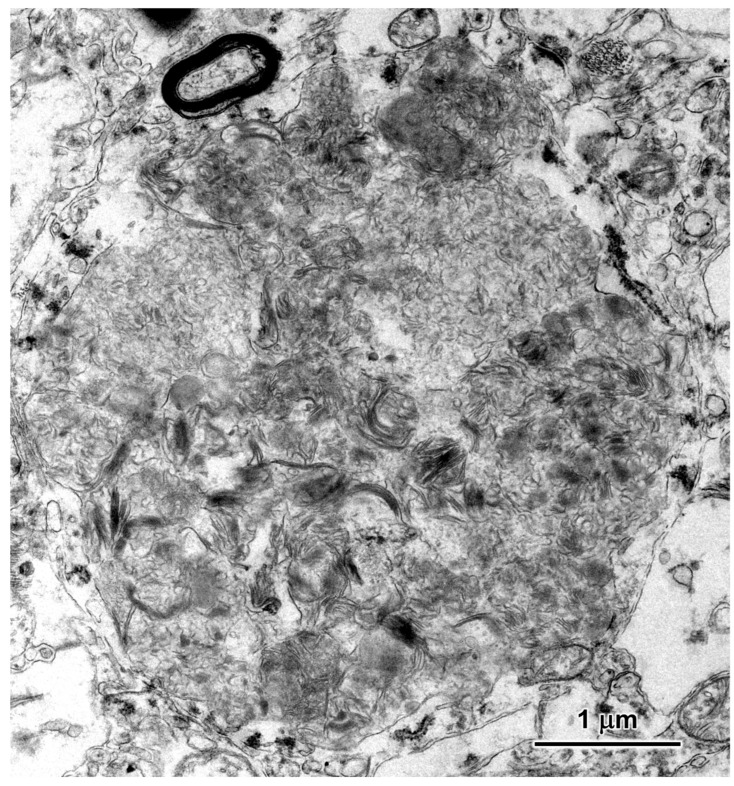
Electron micrograph of a storage body from a cerebral cortical neuron of the proband. A minority of the storage bodies in the cerebral cortex contained a heterogenous mixture of components as shown in this example.

**Figure 12:**
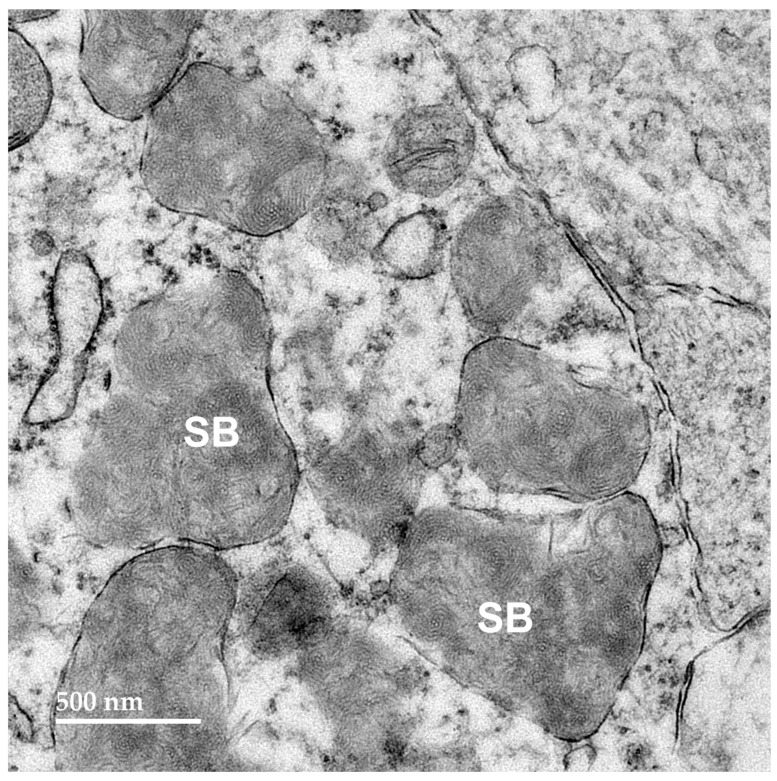
Electron micrograph of a cluster of storage bodies (SB) in a retinal ganglion cell of the proband. The storage bodies have membrane-like contents arranged in fingerprint-like patterns.

**Figure 13:**
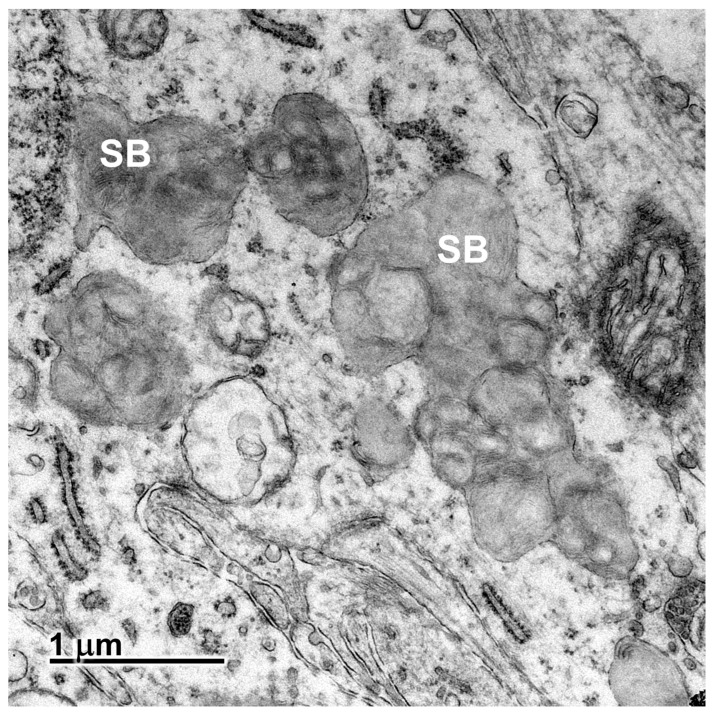
Electron micrograph of a cluster of storage bodies (SB) in the inner retina of the proband. The storage bodies have membrane-like components in relatively random orientations.

**Figure 14:**
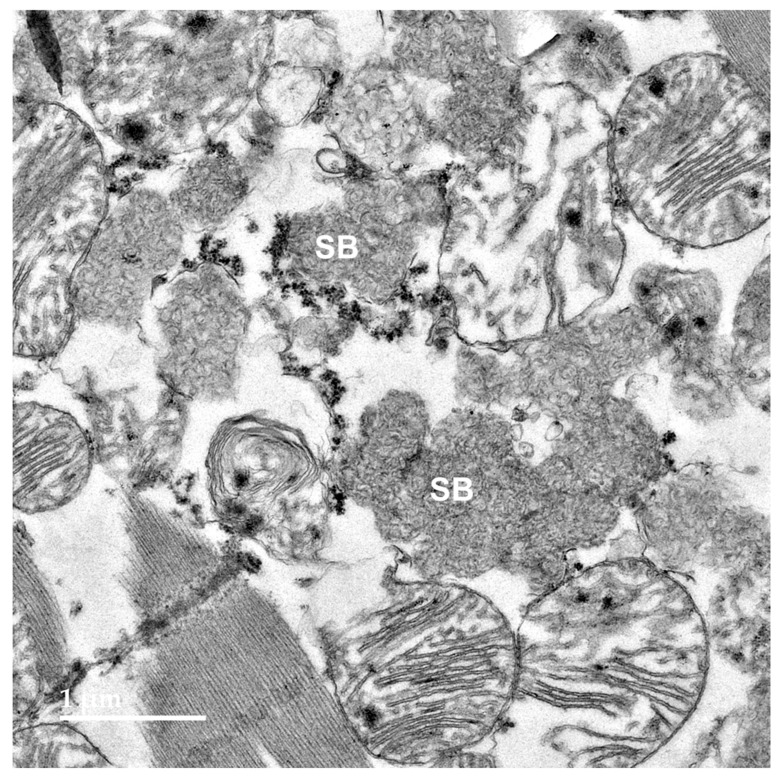
Electron micrograph showing disease-related storage bodies (SB) in the cardiac muscle of the proband.

Electron microscopic examination of the neural tissues of the proband showed that the contents of the disease-related inclusions consisted primarily of membrane-like components (Figure 9, Figure 10, Figure 11, Figure 12 and Figure 13). Within the cerebellar cortex and cerebral cortex, the storage bodies were relatively uniform in appearance, although there were differences in the arrangements of the membranous components between these two areas of the brain (Figure 9 and Figure 10). In a minority of very large storage bodies within cerebral cortical neurons, the arrangements of the membranous contents were more heterogenous (Figure 11). Disease-related inclusion bodies in the retinal ganglion cells contained membrane-like material arranged in fingerprint-like patterns (Figure 12). In other cells of the inner retina, the membrane-like contents of the storage bodies were more randomly arranged (Figure 13). In the cardiac muscles, the storage bodies were interspersed with mitochondria adjacent to the muscle fiber cell nuclei and contained tightly packed vesicular material (Figure 14).

**Figure 15:**
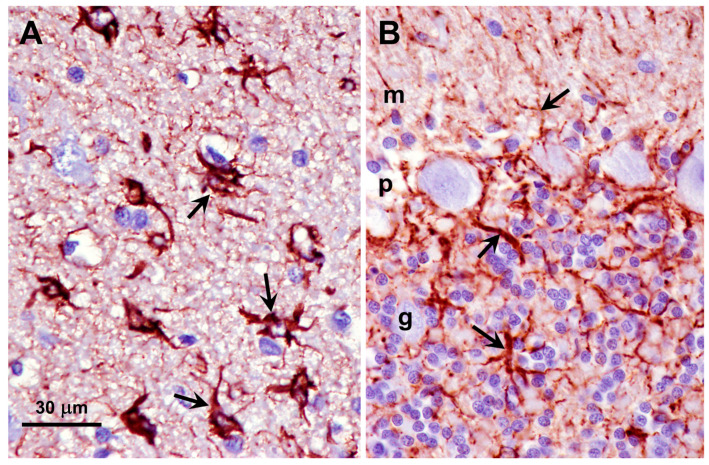
Sections of the cerebral cortex gray matter (**A**) and cerebellar cortex (**B**) from the proband immunostained for GFAP localization (red-brown stain). Activated astrocytes identified by immunolabeling were abundant in the cerebral cortex (arrows in **A**), and processes of activated astrocytes were present in the molecular (m), Purkinje cell (p), and granule cell (g) layers of the cerebellum (arrows in **B**). Bar indicates magnification for both micrographs.

**Figure 16:**
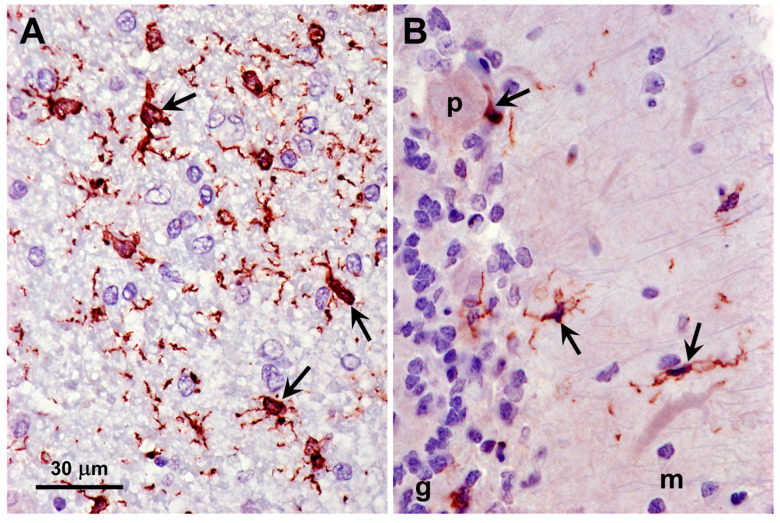
Sections of the cerebral cortex gray matter (**A**) and cerebellar cortex (**B**) from the proband immunostained for Iba1 localization (red-brown stain). Activated microglia identified by immunolabeling were abundant in the cerebral cortex (arrows in **A**). Smaller numbers of activated microglia were present in the molecular (m), Purkinje cell (p), and granule cell (g) layers of the cerebellum (arrows in **B**). Bar indicates magnification for both micrographs.

Activation of glia, indicative of neuroinflammation, occurs in many neurodegenerative disorders, including most NCLs. Activated astrocytes, identified by immunolabeling for GFAP, were abundant in both the cerebral cortex and cerebellar cortex of the proband (Figure 15). Activated microglia, identified by immunolabeling for Iba1, were abundant throughout the gray matter of the cerebral cortex and were also present to a lesser degree in all layers of the cerebellar cortex of the proband (Figure 16).

### 3.3. Molecular Genetics

The proband was one of two affected dogs in a litter of nine that were born to healthy parents, consistent with an autosomal recessive mode of inheritance. The whole-genome sequence data of the proband, 3 healthy littermates, and the sire were analyzed in conjunction with similar data generated with DNA from 383 dogs of other breeds that included healthy dogs and dogs with a variety of diseases. These 383 whole-genome sequences served as controls and allowed us to screen for variant alleles in the proband that were rare or absent in the control population. Based on the proband’s pedigree, we hypothesized that the disease was inherited as an autosomal recessive trait. Whole-genome sequences of the proband, the unaffected littermates, and the unaffected sire were compared to identify variants that were homozygous in the proband, heterozygous in the sire, and either heterozygous or absent in the unaffected littermates.

Based on an autosomal recessive model of inheritance, variants identified with WGS analysis were filtered through a series of sequential steps and are summarized in Table 1. Relative to the Dog10K_Boxer_Tasha reference genome, the proband was found to be homozygous for 20,399 variants. Of the 5 PBDGs of the family, the proband was the only one that was homozygous for 2607 of these variants. Since the sire of the proband did not exhibit disease signs, he was hypothesized to be heterozygous for the causal variant. Of the 2607 variants for which the proband was the only homozygous PBDG, 2403 were heterozygous in the sire. Our control cohort of 387 dogs consisted of the four unaffected PBDGs and 383 dogs of other breeds from which we had generated WGS data. The disease signs exhibited by the proband were distinct from those of any other dog in the control cohort. Therefore, of the 2403 variants, only 41 that were homozygous exclusively in the proband were considered as potentially causal candidates. To further narrow down the potential causal variants, any that were present in the control cohort were excluded, leaving 10 variants for further consideration. These variants are listed in Table 2.

**Figure 17:**
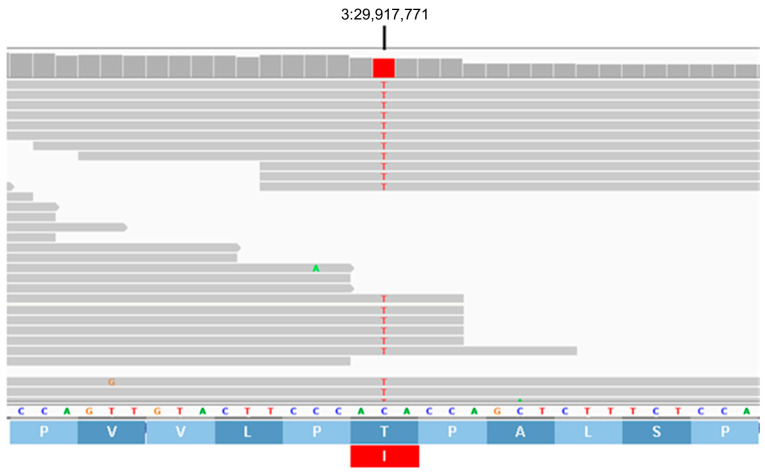
Screenshot of the proband’s whole-genome sequence reads aligned to the reference sequence in the vicinity of position 29,917,771 on chromosome 3 (red bar at top), as viewed with the Integrative Genomics Viewer. The variant T is highlighted in red. The reference DNA sequence is shown along with the predicted AP3B1 protein amino acid sequence. The codon change from ACA to ATA predicts an amino acid change from threonine (T) to isoleucine (I).

**Figure 18:**
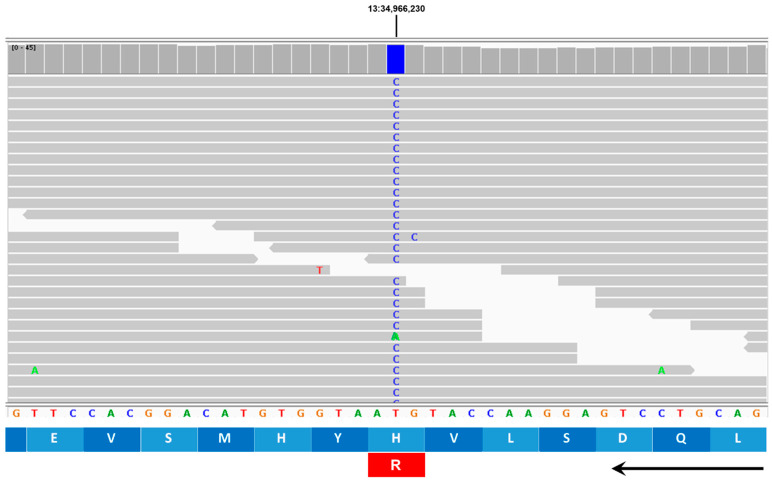
Screenshot of the proband’s whole-genome sequence reads aligned to the reference sequence in the vicinity of position 34,966,230 on chromosome 13 (blue bar at top), as viewed with the Integrative Genomics Viewer. The variant C is highlighted in blue. The reference DNA sequence is shown along with the predicted TRAPPC9 protein amino acid sequence (arrow indicates amino acid sequence from amino to carboxy terminal direction). The codon change from GTA to GCA predicts an amino acid change from histidine (H) to arginine (R).

**Figure 19:**
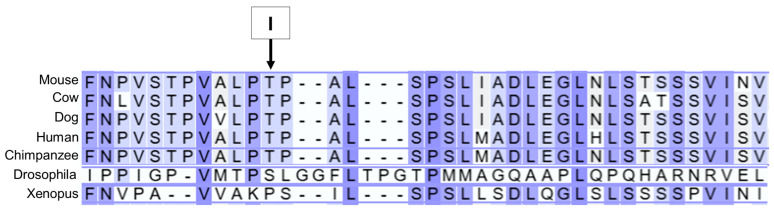
AP3B1 amino acid sequence alignments for multiple species surrounding residue 832 in the canine reference sequence (arrow). In all mammalian species the residue at this position is threonine, whereas isoleucine is predicted at this position in the proband. The amino acid sequences of Drosophila and Xenopus differ significantly from the mammalian sequences.

**Figure 20:**
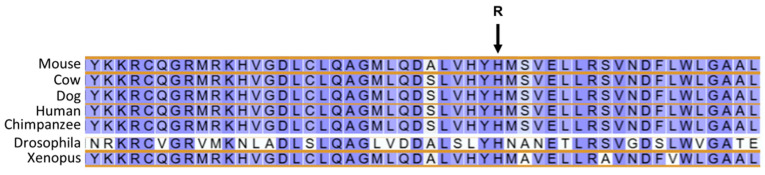
TRAPPC9 amino acid sequence alignments for multiple species surrounding residue 225 in the canine reference sequence (arrow). In all species the residue at this position is histidine, whereas the proband was homozygous for arginine at this position.

Based on reviews of published information on the functions of the proteins encoded by these 10 genes, the variants in *AP3B1* and *TRAPPC9* appeared most likely to be associated with the disease phenotype of the proband. The structures of these genes are annotated in Ensembl with accession numbers ENSCAFG00845008344 (*AP3B1*) and ENSCAFG00000001181 (*TRAPPC9*). The genotypes at the variant loci were confirmed by examining the WGS data for these regions with the Integrative Genomics Viewer (Figure 17 and Figure 18). The amino acids at the equivalent positions across diverse mammalian species matched those in the canine reference sequence (Figure 19 and Figure 20), suggesting that the amino acids at these positions have highly conserved functional roles. Therefore, all 42 of the PBDGs from which we had DNA samples and phenotype information were genotyped at these two variant loci (Table 3). Of the 41 unaffected dogs, three were homozygous for the *AP3B1* variant and only one was homozygous for the *TRAPPC9* variant. The proband was the only dog homozygous for both variants. Four dogs, including the sire and two littermates of the proband were heterozygous for both variants (Table 4). Chi-square analysis indicated that the probability that the genotype-phenotype associations were due to chance was less than one in over 100,000 (Chi-square = 468, *p* < 10^−5^).

## 4. Discussion

The proband PBDG presented with a progressive neurological disorder with signs similar to those of dogs with various forms of NCL [1]. Based on the dog’s pedigree, the disorder appeared to be inherited as an autosomal recessive trait. Postmortem examination of brain, retina, and heart revealed abundant accumulations of autofluorescent storage material with ultrastructural appearances similar to those of disease-related storage bodies that accumulate in both human and canine NCLs [1,2,16,33,37,38,39,40,41]. However, no homozygous variants in the WGS of the proband were found in any of the known or previously proposed NCL genes. Filtering the DNA sequence variants in the proband to only those that were uniquely homozygous in the proband relative to its sire and three unaffected littermates and 383 unrelated dogs of other breeds identified 41 potential causal variants. It was hypothesized that the causal variant would be unique to the PBDG breed. Thirty-one of the 41 candidate variants were present in the heterozygous state in dogs of other breeds, leaving 10 candidates as possible causal variants. Based on what has been reported regarding the functions of the proteins encoded by these genes, missense variants in two of them, *AP3B1* and *TRAPPC9* were considered to most likely underlie the disorder in the proband.

In order to further investigate the association between these variants and the disorder in the breed, additional PBDGs were recruited. PBDGs are uncommon in Sweden where the proband resided, so other than the littermates and sire, very few other dogs of this breed could be recruited from Sweden. Therefore, additional PBDGs were recruited from France where the breed is more common and where the ancestors of the Swedish dogs originated. No other PBDGs from either country that we were able to recruit exhibited disease signs similar to those of the proband. Therefore, it appears that the disease in the proband is quite rare in the breed, if not unique. Genotyping the unaffected PBDGs for the candidate *AP3B1* and *TRAPPC9* variants identified three other dogs that were homozygous for the *AP3B1* variant and one other that was homozygous for the *TRAPPC9* variant. None of these dogs exhibited disease signs similar to those of the proband. In addition, significant proportions of the unaffected dogs were heterozygous for each of these variants (41% and 34%, respectively). However, only five of them were heterozygous for both variants. Two of these were littermates of the proband and another was the proband’s sire. Excluding this family, combined heterozygosity for the two variants occurred in only two of 34 dogs. The data are consistent with the hypothesis that the disease develops only in dogs homozygous for both variants. If this is the case, affected dogs could only result from breeding unaffected dogs if both sire and dam were heterozygous for both variants. The rarity of such a genotype in the breed is consistent with the fact that we were not able to identify any other affected dogs among those not closely related to the proband. The hypothesis that the disorder results from the combined effects of the AP3B1 and TRAPPC9 amino acid substitutions can be confirmed if any additional affected PBDGs are identified. Screening additional unaffected PBDGs for these variants and genotyping new cases would be needed to confirm this hypothesis. Although the probability of additional affected dogs being generated appears to be low, because the PBDG is an uncommon breed with a small breeding pool, there is a potential for the risk variants to become more common if surveillance is not undertaken, particularly since each of the two variants is relatively common individually in the cohort of PBDGs that we were able to evaluate.

**Figure 21:**
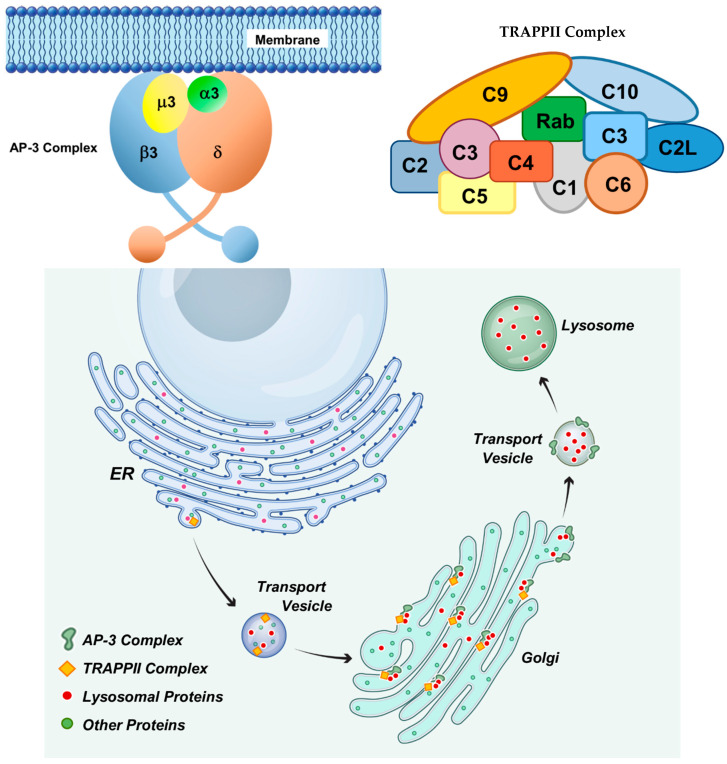
Diagram illustrating the roles of the proteins encoded by *AP3B1* and *TRAPPC9* in transporting proteins to lysosomes. *AP3B1* encodes the b subunit of the AP-3 protein complex that consists of 4 subunits. *TRAPPC9* encodes the C9 subunit of the TRAPPII protein complex that consists of at least 11 subunits. Both protein complexes mediate transport from the endoplasmic reticulum through the Golgi apparatus to lysosomes. Synergistic interactions between the AP-3 and TRAPPII complexes mediated by the β and C9 subunits may impair delivery of proteins from the Golgi to lysosomes.

The neurological signs and tissue pathology in the proband were similar to those of canine NCLs, which are types of lysosomal storage disorders [1,16,33,36,37,38,42,43]. Thus, it appeared likely that the disorder in the proband was the result of lysosomal dysfunction. The proteins encoded by both *AP3B1* and *TRAPPC9* play key roles in the delivery of protein constituents to lysosomes (Figure 21). They are each components of protein complexes that mediate transport of proteins from the endoplasmic reticulum, through the Golgi apparatus, to lysosomes [44,45,46,47,48,49,50,51,52,53,54,55]. The AP3B1 protein is a component of the heterotetrametric adaptor protein complex AP-3 that plays an important role in the sorting and vesicular transport of proteins from the Golgi apparatus to lysosomes (Figure 21) [44,45,50,51,52]. This complex has been localized to the Golgi apparatus and to vesicles transporting proteins to lysosomes. The TRAPPC9 protein is a subunit of a large muti-subunit complex (Transport Protein Particle II, TRAPPII) involved in mediating vesicle transport from the endoplasmic reticulum to the Golgi apparatus, within the Golgi apparatus, and exit of transport vesicles from the Golgi apparatus (Figure 21) [47,49,53]. Dogs that were homozygous for either the *AP3B1* or *TRAPPC9* variant alone did not exhibit neurological signs that characterized the disease in the proband. The fact that the disease only developed in the dog homozygous for both variants suggests that the protein complexes that contain these two proteins function together in a manner that involves interaction of the *AP3B1*- and *TRAPPC9*-encoded proteins. The findings of this study suggest that the AP-3 and TRAPPII complexes interact within the Golgi via the AP3B1 and TRAPPC9 proteins to facilitate transport of proteins to lysosomes. Impairment of this interaction as a consequence of the combined amino acid changes in these two proteins may be the cause of lysosomal dysfunction and associated accumulation of lysosomal storage bodies in the proband. Further research will be necessary to determine the nature of the interaction between the two protein complexes that is suggested by the findings of this study.

In some forms of NCL where the causal DNA sequence variant clearly affects lysosomal function, a major component of the lysosomal storage material is the subunit c protein of mitochondrial ATP synthase [42,56,57,58,59,60]. That this same protein accumulated in the storage bodies of the affected PBDG supports the hypothesis that lysosomal function was impaired. The storage bodies in the proband also exhibited autofluorescent properties that are characteristic of the NCLs. The similarity between the PBDG disorder and the canine and human NCLs suggests that it may be appropriate to classify this disease as yet another NCL disorder. However, no human neurological disorders have yet been associated with combined variants in *AP3B1* and *TRAPPC9*.

Mutations in *TRAPPC9* alone have been associated with autosomal-recessive intellectual disability, developmental delay, autism, seizures, brain structure abnormalities, brain atrophy, and microcephaly in human subjects, with significant variability in the phenotypes of the disorders [61,62,63,64,65,66]. Tissues from these patients do not appear to have been evaluated for the accumulation of lysosomal storage bodies, but in cells cultured from a subject with a homozygous *TRAPPC9* missense variant and severe developmental delay, intracellular vesicular trafficking was impaired [61]. This is consistent with impaired transport of lysosomal enzymes from the Golgi apparatus. Unlike the NCLs, the human disorders associated with *TRAPPC9* variants do appear to progress after early childhood.

Mutations in *AP3B1* have been associated with type 2 Hermansky–Pudlak syndrome in human subjects [67,68,69,70]. The disorder is characterized by oculocutaneous albinism, visual impairment, bleeding diathesis, and pulmonary fibrosis associated with impaired vesicular sorting of proteins to platelet dense granules, melanosomes, and lysosomes [67]. As in the NCLs, the disorder is characterized by accumulation of ceroid lipofuscin [71,72]. However, unlike the NCLs, the *AP3B1* disorder has not been associated with progressive neurodegeneration.

Independently, deleterious variants in *TRAPPC9* and *AP3B1* do not result in NCL-like disorders. Thus, the phenotype associated with the combined variants in these two genes in the affected dog is different in many respects than the effects of deleterious variants in either gene alone. This study suggests that variants may alter the interactions between protein complexes significantly only when both interacting partners are modified. In this case, the combined effect appears to alter lysosomal function, resulting in the accumulation of lysosomal storage bodies. In some NCLs with pathology similar to that of the PBDG proband, the underlying cause also appears to be the result of impaired intracellular transport of proteins to lysosomes [73,74,75].

## 5. Conclusions

An NCL-like disorder in a PBDG dog appears to be the result of impaired trafficking of proteins to lysosomes due to a combination of missense variants in *TRAPPC9* and *AP3B1*.

## Figures and Tables

**Table 1:** WGS variant sequential filtering steps.

Filtering Steps	Number of Variants
Proband was homozygous relative to the Dog10K_Boxer_Tasha reference	20,399
Proband was the only homozygous PBDG within its family	2607
Sire was heterozygous	2403
Proband was the only homozygote among 387 other dogs *	41
Proband uniquely homozygous and no heterozygotes in control cohort **	10

* Excluding proband and including 4 unaffected PBDGs (sire and 3 littermates); ** Control cohort excluding unaffected sire and 3 littermates (*n* = 383).

**Table 2:** Candidate disease variants.

Chr.	Position ^1^	Ref. ^2^	Alt ^3^	Effect	AA Change	Gene ID
2	70,241,214	T	C	Missense	C340G	*ZNF683*
2	70,429,672	A	AC	3′UTR	-	*PDIK1L*
3	29,917,771	C	T	Missense	T832I	*AP3B1*
5	61,470,177	G	A	Stop Gained	W373 *	*PER3*
5	61,564,260	A	T	Missense	F190I	*TNFRSF9*
8	67,681,637	C	T	5′UTR	-	*EML1*
13	34,966,230	T	C	Missense	H225R	*TRAPPC9*
13	37,186,500	G	A	Missense	R870C	*MROH6*
27	5,344,292	CTCTCCG	C	5′UTR	-	*PRMT8*
27	6,171,476	CG	C	3′UTR	-	*FGF23*

^1^ Positions based on Dog10K_Boxer_Tasha genome assembly. ^2^ Reference allele. ^3^ Proband allele. Highlighted candidates were deemed most likely to be associated with the disease.

**Table 3:** Genotype distribution in a cohort of unaffected PBDGs.

	Number *	
Genotype **	*AP3B1*	*TRAPPC9*
V/V	3 ^#^	1 ^##^
R/V	17	14
R/R	21	26

* Includes sire and 3 littermates of proband; ** V:variant; R:reference; ^#^ All 3 unrelated to proband; ^##^ Littermate of proband.

**Table 4:** Distribution of heterozygous genotypes in the cohort of all unaffected PBDGs.

Heterozygous For:	Number *
*AP3B1* Only	12
*TRAPPC9* Only	9
Both	5 **

* Includes sire and 3 littermates of proband; ** Of the 5, one was the sire and two were littermates of proband and the other two were unrelated dogs.

## Data Availability

Sequence data generated for this study are available in the NCBI Sequence Read Archive with the identification numbers provided in Supplementary File S1.

## Supplementary Materials

The following supporting information can be downloaded at: https://www.mdpi.com/article/10.3390/genes16111370/s1, Supplementary File S1. Whole-genome sequence BioSample IDs for whole-genome sequences deposited the NCBI Sequence Read Archive. Supplementary File S2. Blood test results for the proband prior to euthanasia.

## Abbreviations

The following abbreviations are used in this manuscript:

PBDG	Petit Bleu de Gascogne
NCL	Neuronal ceroid lipofuscinosis
MRI	Magnetic resonance imaging
