# Supplementary material for: Canine Neuronal Ceroid Lipofuscinosis-like Disorder Associated with Sequence Variants in AP3B1 and TRAPPC9
Source: Genes (Basel). 2025 Nov 11;16(11):1370. doi: 10.3390/genes16111370 (PMC12651985; doi:10.3390/genes16111370)
Supplement: Supplementary file 1 [file genes-16-01370-s001.zip › Supplementary File 2- Blood Test Results.pdf]

Supplementary Data: Blood Test Results

2021-05-20

**Analyte (Method/Lab)**	**Result**	**Units**	**Reference Interval**
Plasma AST (VetScan)	0.8	µkat/L	0.20 – 0.80
Plasma CK (VetScan)	2.3	µkat/L	0.30 – 3.30
Plasma Magnesium (VetScan)	0.82	mmol/L	0.74 – 0.99

2021-05-20 — Bile acids stimulation test

**Analyte (Method/Lab)**	**Result**	**Units**	**Reference Interval**
Basal bile acids	1.7	µmol/L	0 – 20
Post-stimulation bile acids	23.9	µmol/L	—

2021-04-07

**Analyte (Method/Lab)**	**Result**	**Units**	**Reference Interval**
IDEXX cTLI (canine trypsin-like immunoreactivity)	14.6	µg/L	8.50 – 35.00
IDEXX Folate	17.7*	nmol/L	21.10 – 54.00
IDEXX Vitamin B12 (Cobalamin)	314	pmol/L	173.00 – 599.00
IDEXX Cortisol	165.0*	nmol/L	24.80 – 124.20
IDEXX Spec cPL (canine)	<30	µg/L	< 200.00

2021-03-25

**Analyte (Method/Lab)**	**Result**	**Units**	**Reference Interval**
Blood Hemoglobin (Hb)	17.2	g/dL	13.10 – 20.50
Hematocrit (HCT)	49.7	%	37.30 – 61.70

Erythrocytes (RBC)	7.64	×10^12/L	5.65 – 8.87
Mean Corpuscular Volume (MCV)	65.1	fL	61.60 – 73.50
Mean Corpuscular Hemoglobin (MCH)	22.5	pg	21.20 – 25.90
Mean Corpuscular Hemoglobin Concentration (MCHC)	34.6	g/dL	32.00 – 37.90
Reticulocytes	10.7	K/µL	10.00 – 110.00
Reticulocyte hemoglobin (RET-HGB)	23.5	pg	22.30 – 29.60
Leukocytes (WBC)	11.92	×10^9/L	5.05 – 16.76
Differential — Neutrophils, bands	—	10^9/L	0.00 – 0.30
Differential — Neutrophils, segmented	7.67	10^9/L	3.00 – 11.50
Differential — Eosinophils	0.77	10^9/L	0.10 – 1.30
Differential — Basophils	0.01	10^9/L	0.00 – 0.10
Differential — Lymphocytes	2.85	10^9/L	1.00 – 4.80
Differential — Monocytes	0.62	10^9/L	0.20 – 1.40
Differential — Other cells	—	10^9/L	—
Erythrocyte morphology	Numerous echinocytes	—	—
Platelets (PLT)	321	K/µL	148.00 – 484.00
Mean Platelet Volume (MPV)	10.8	fL	8.70 – 13.20
Plateletcrit (PCT)	0.35	%	0.14 – 0.46
Plasma ALT (cobas)	0.92	µkat/L	0.15 – 1.60
Plasma ALP (cobas)	0.6	µkat/L	0.10 – 1.50
Plasma Albumin (cobas)	41	g/L	30.00 – 45.00
Plasma Bilirubin (cobas)	<1.7	µmol/L	< 3.20
Plasma Calcium (cobas)	2.80	mmol/L	2.40 – 3.00

Plasma Phosphorus (cobas)	1.16	mmol/L	0.70 – 1.90
Plasma Glucose (cobas)	5.5	mmol/L	3.70 – 6.60
Plasma Creatinine (cobas)	76	µmol/L	42.00 – 110.00
Plasma Total Protein (cobas)	67	g/L	49.00 – 71.00
Plasma Urea (cobas)	5.9	mmol/L	2.70 – 8.70
Plasma Sodium (cobas)	142	mmol/L	138.00 – 149.00
Plasma Potassium (cobas)	4.1	mmol/L	3.40 – 4.80

Symbols: ‘—’ = not applicable/not reported; ‘<’ = below assay reporting threshold.
